# Estrogen Receptor Beta Prevents Signet Ring Cell Gastric Carcinoma Progression in Young Patients by Inhibiting Pseudopodia Formation via the mTOR–Arpc1b/EVL Signaling Pathway

**DOI:** 10.3389/fcell.2020.592919

**Published:** 2021-01-21

**Authors:** Xingzhou Wang, Xuefeng Xia, En Xu, Zhi Yang, Xiaofei Shen, Shangce Du, Xiaotong Chen, Xiaofeng Lu, Wei Jin, Wenxian Guan

**Affiliations:** ^1^Department of General Surgery, Affiliated Drum Tower Hospital of Nanjing University Medical School, Nanjing, China; ^2^Department of Neurosurgery, Affiliated Drum Tower Hospital of Nanjing University Medical School, Nanjing, China; ^3^Department of General Surgery, Drum Tower Clinical Medical College of Nanjing Medical University, Nanjing, China; ^4^Comprehensive Cancer Centre of Drum Tower Hospital, Medical School of Nanjing University and Clinical Cancer Institute of Nanjing University, Nanjing, China

**Keywords:** signet ring cell gastric carcinoma, estrogen receptor beta (ERß), pseudopodia, clinicopathologic analysis, mTOR

## Abstract

Signet ring cell gastric carcinoma (SRCGC) is a poorly differentiated malignancy, and can be highly dangerous in the progression stage. There is a higher male to female ratio among patients with signet ring cell carcinoma as compared to patients with non-SRCGC. ERβ has been found to express in stomach adenocarcinoma, but how it affects tumor progression remains unclear. Here, we studied estrogen receptor beta (ERβ) to explore the role of sex-associated factors in SRCGC. We analyzed the clinicopathological statistics of patients with SRCGC, and conducted a series of *in vitro* experiments. Immunohistochemistry showed that patients with low ERβ expression were at risk of poor prognosis and higher T stage. *In vitro* assays indicated that ERβ might prevent SRCGC progression by inhibiting cell proliferation and invasiveness and by promoting anoikis. Western blotting and quantitative RT-PCR proved that the mTOR–Arpc1b/EVL signaling pathway might participate in the negative regulatory role of ERβ. In conclusion, our findings show that ERβ might inhibit the malignancy of signet ring cells in patients with SRCGC, indicating that ERβ might be a potential target in adjuvant treatment.

## Introduction

Signet ring cell gastric carcinoma (SRCGC) is a unique, rare form of poorly differentiated gastric adenocarcinoma. Patients with SRCGC comprise 11–26% of all patients with gastric cancer (Taghavi et al., 2012; Peng et al., 2014); many of these patients are diagnosed in the progressive stage or even late stage. Although patients with early SRCGC often have relatively good prognosis compared to patients with the typical type of stomach adenocarcinoma (Imamura et al., 2016), it is more aggressive and malignant as it develops to the progressive stage, and predicts worse overall survival (Kwon et al., 2014; Kao et al., 2019). Moreover, SRCGC is more invasive and can invade the serosal layer, and due to its low-attachment characteristic, the cancer cells are more capable of detaching from the primary lesion to other cavities, such as the peritoneal cavity, and forming distant metastatic niches. Clinical statistics have shown that patients with SRCGC tend to be female and young (Kao et al., 2019), which implies that estrogen and its receptors could play an essential role in SRCGC.

Estrogen is one of the most common hormones in the interior milieu, participating in the development of the female reproductive system and the regulation of the secondary sex characteristics. Beyond its physiological function, it is involved in the progression of malignancies, such as breast cancer (Zhou et al., 2015; Jiang et al., 2018) and ovarian cancer (Ribeiro and Freiman, 2014; Hodgkinson et al., 2018), by binding to estrogen receptors (ERs) and activating the downstream signaling pathways to regulate the expression of many other genes. Even though there is no significant difference in stomach adenocarcinoma morbidity between male and female patients, we were interested in whether estrogen and its receptors have an influence on SRCGC.

ERs are composed of membrane receptors (mERs), such as GPER (G protein–coupled ER), and nuclear receptors, which include ER alpha (ERα, ESR1) and ER beta (ERβ, ESR2). In general, ERα is the main receptor for estrogen. However, in stomach adenocarcinoma, ERβ is expressed dominantly instead of ERα (Matsuyama et al., 2002; Takano et al., 2002). Several studies have directly or indirectly confirmed the suppressive effect of ERβ in stomach adenocarcinoma progression (Wang et al., 2007; Xu et al., 2010). Consequently, ERβ is thought to be suppressive of SRCGC progression, and its loss of expression may be linked with prognosis of SRCGC.

Previously, our research team focused on the mechanism of how stomach adenocarcinoma cells gain anoikis-resistant ability and metastasize to the distant organs (Du et al., 2018). Stomach adenocarcinoma is derived from gland epithelial cells, i.e., the tumor cells are anchorage-dependent initially and that are not resistant to anoikis, a programmed cell death triggered by cell detachment from the extracellular matrix or loss-of-junction from other cells. We have also confirmed previously that these cells have the potential to invade and migrate to the distant organs, and scanning electron micrographs show the formation of pseudopodia in these cells (Du et al., 2018). SRCGC cells have *CDH1* mutation and are relatively anoikis-resistant, surviving without extracellular matrix. Therefore, we used immunohistochemistry (IHC) to explore ERβ expression in young patients with SRCGC, and found that ERβ downregulation or ectopic expression in the cytoplasm could lead to worse prognosis of SRCGC. *In vitro* assays confirmed that ERβ could promote anoikis and decrease cell invasiveness and proliferation to prevent cancer metastasis. The pseudopodia formation assay showed that the loss of ERβ could lead to pseudopodia formation and improve invasiveness. We noticed that actin-related protein complex subunit 1b (Arpc1b) and Enah/Vasp-like (EVL), as downstream products regulated by ERβ, are involved in cytoskeleton remodeling (Kumar et al., 2015). Therefore, we detected the mRNA and protein level of Arpc1b and EVL and concluded that ERβ might affect tumor development through the mTOR–Arpc1b/EVL signaling pathway.

## Materials and Methods

### Clinical Tissue Samples and IHC

SRCGC is defined as >50% signet ring cells in the mucosa layer. After screening, 42 patients diagnosed with SRCGC and who had undergone standard D2 resection or palliative surgery between August 2010 and July 2019 at Nanjing Drum Tower Hospital and who were aged <45 years were included in our research group. All the female patients were confirmed to have regular menstrual cycle. Patient information was routinely followed-up every 6 months. Patients diagnosed with mixed adenocarcinoma with not more than 50% signet ring cells were excluded. Patients who had received adjuvant therapy, including chemotherapy, radiotherapy, or any other anti-tumor treatment (such as endoscopic submucosal dissection or endoscopic mucosal resection) before surgery were also excluded. None of the patients had been diagnosed with gastric cancer or other malignancies beyond SRCGC. An experienced doctor collected and checked the clinicopathological statistics.

Once removed from the patients, the collected tumor tissues were treated with formalin and paraffin. The paraffin blocks were stored correctly before use. Then, the samples were cut into sections, de-paraffinized, and rehydrated. IHC assay was performed using recombinant rabbit monoclonal antibody against ERβ (ab288, 1:200 dilution, Abcam, United States). After heating in 10 mM citrate buffer at 98°C for 25 min, the sections were cooled to 25°C for retrieval, and were incubated in 0.3% H_2_O_2_ for 30 min to deactivate endogenous peroxidases. Then, the sections were incubated with anti-ERβ antibody overnight at 4°C after 60-min blocking with 5% goat serum dissolved in phosphate-buffered saline (PBS). All sections were stained with diaminobenzidine solution and counter-stained with hematoxylin. After staining, all sections were evaluated by two senior pathologists independently, and the IHC results were recorded in a blinded manner. The score was assigned according to the percentage of positively stained cells (0 = 0–5% of cells, 1 = 6–25% of cells, 2 = 26–50% of cells, 3 = 51–75% of cells, 4 = 76–100% of cells) and staining intensity (0 = no staining, 1 = weak staining, 2 = moderate staining, 3 = strong staining). The final score equaled the sum of both scores. All materials and their usage in this project were reviewed and approved by the Nanjing Drum Tower Hospital Review Board.

### Materials

The primary antibodies were against Rac1 (ab33186, Abcam), GAPDH (ab181602, Abcam), phosphorylated (p)-mTOR (5536T, Cell Signaling Technology, United States), mTOR (2983T, Cell Signaling Technology), ERβ (ab3576, Abcam), and cortactin (ab33333, Abcam). The secondary antibodies were horseradish peroxidase (HRP)-linked anti-rabbit immunoglobulin G (IgG) (7074S, Cell Signaling Technology), HRP-conjugated AffiniPure goat anti-mouse IgG (H++L) (SA00001-1, Proteintech, United States), and goat anti-mouse IgG H&L (Alexa Fluor^®^ 488) (ab150113, Abcam). 17-β-Estradiol and tamoxifen was purchased from MedChemExpress to activate or modulate ER, respectively.

### Cell Culture

The human SRCGC cell line NUGC-4 was a kind present from Dr. Ruping Wang (Nanjing, China). Once received, the cells were passaged for one generation, and then STR (short tandem repeat) authentication was performed immediately by Genetic Testing Biotechnology (Suzhou, China). After authentication, the cells were incubated at 37°C in 5% CO_2_ in RPMI 1640 medium containing 10% fetal bovine serum (Biological Industries, Israel). The medium was renewed every 2 days and passaged at 80–90% density. Suspended tumor cells were cultivated using Costar^®^ 6-well Clear Flat Bottom Ultra-Low Attachment Multiple Well Plates (3471, Corning, United States).

### Asian Cancer Research Group (ACRG) Cohort Analysis

We enrolled the gene expression datasets of the ACRG cohort (GSE62254) to analyze the association between *ESR2* expression level and the prognosis of patients with SRCGC. The processed microarray profiles and clinical data of the ACRG cohort (*n* = 300) were downloaded from the Gene Expression Omnibus (GEO) database through the R package GEOquery. Using the R package survminer, the patients were automatically divided into *ESR2* low and high expression (ERβ^low^ and ERβ^high^, respectively) groups according to *ESR2* expression by best cutoff, and were used to construct the Kaplan–Meier survival curves.

### Flow Cytometry

Flow cytometry was mainly used to analyze the anoikis rate and cell cycle of NUGC-4 cells under suspension condition. The collected cells were incubated with FITC–Annexin V Apoptosis Detection Kit II (556570, BD Bioscience, United States) to detect the apoptosis rate. To detect the cell cycle, tumor cells were synchronized by serum withdrawal for 48 h. Then the cells were incubated using RPMI-1640 with 10% FBS and different treatment for 24 h. After that, pre-cooled absolute ethanol was used to fix and penetrate. The cell cycles were investigated using Cell Cycle Detection Kit (KGA512, Keygen, Nanjing, China). The fluorescence results were detected and obtained by a BD Accuri^TM^ C6 Plus Cell Analyzer (BD Bioscience). The final results were analyzed using FlowJo 10.4 (BD Bioscience).

### Protein Extraction and Immunoblotting

The pre-treated NUGC-4 cells were scraped and lysed with radioimmunoprecipitation assay (RIPA) buffer containing 1:100 complete^TM^ Protease Inhibitor Cocktail (Roche, United States) in 1.5 ml EP tubes on ice for 20 min. Then, the lysed samples were centrifuged at 12,000 rpm for 20 min at 4°C. After centrifugation, the supernatants were transferred to clean EP tubes and mixed with 1:4 volume 5 × sodium dodecyl sulfate–polyacrylamide gel electrophoresis (SDS-PAGE) loading buffer (BioSharp Inc., Nanjing). The samples were placed in boiling water for 5 min to ensure protein denaturation. Protein was quantified using bicinchoninic acid (BCA) reagent (Keygen). Subsequently, equal amounts of proteins (15 μg) from each sample were added to SDS-PAGE to separate the target protein, and then transferred onto polyvinylidene fluoride (PVDF) blotting membranes (GE Healthcare, Germany). The polyacrylamide gels were prepared in advance using a PAGE Gel Fast Preparation Kit (Epizyme Inc., Nanjing). Next, the membranes were blocked with 5–10% skim milk for 1 h, then cut into strips and incubated with the primary antibodies overnight and subsequently with the secondary antibodies for 1 h. Protein bands were visualized according to the instructions of the PageRuler pre-stained protein ladder (Thermo Fisher Scientific, United States).

### RNA Extraction and Complementary DNA (cDNA) Synthesis

In brief, total RNA was extracted from the cells using TRIzol (Sigma, United States). After extraction, precipitation, and washing by chloroform, isopropanol, and 75% ethanol diluted with DEPC (diethylpyrocarbonate) water (BeyoTime, Nantong, China), the extractions were finally diluted with DEPC water. The concentration and purity of the samples were detected by BioDrop μLITE+ (BioDrop, United Kingdom). The RNA samples were then reverse-transcribed to cDNA using HiScript III RT SuperMix for qPCR (Vazyme Biotech Co., Ltd., Nanjing, China). The cDNA synthesis was carried out following the manufacturer’s instructions.

### Real-Time Quantitative PCR (qRT-PCR)

The target and internal reference gene expression at mRNA level was detected using the comparative threshold cycle (2^–ΔΔ*Ct*^) method by detecting SYBR Green fluorescence radiation on a ViiA^TM^ 7 Real-Time PCR System (Applied Biosystems, United States). The primers were designed in our laboratory and were synthesized by TSINGKE Biological Technology Inc., (Nanjing, China). The final reaction volume for the analysis of target expression was 10 μL: 0.2 μL forward primer and 0.2 μL reverse primer, 5 μL SYBR Green, 1 μL cDNA sample, and 3.6 μL DEPC water. The cycling conditions were 95°C for 30 s, followed by 40 cycles at 95°C for 3 s, 60°C for 10 s at the PCR stage, and finally 95°C for 15 s, 60°C for 60 s, and 95°C for 15 s at the melt curve stage. All runs included one negative DNA or cDNA control consisting of RNase-free water. Relative gene expression at mRNA level was quantified using an internal reference gene in each sample, and the final results were obtained with ViiA^TM^ 7 Software (Applied Biosystems). Table 1 lists the sequences of the primers used. The data were analyzed using GraphPad Prism 7.0 (GraphPad, United States).

**TABLE 1:** Primer used in quantitative real-time PCR assays.

Primer	Sequences (5′→ 3′)
GAPDH-F	TGCACCACCAACTGCTTAGC
GAPDH-R	GGCATGGACTGTGGTCATGAG
β-actin-F	CATGTACGTTGCTATCCAGGC
β-actin-R	CTCCTTAATGTCACGCACGAT
ESR2-F	GGATGAGGGGAAATGCGTAG
ESR2-R	GGCAATCACCCAAACCAAAG
ARPC1B-F	CAAGGACCGCACCCAGATT
ARPC1B-R	TGCCGCAGGTCACAATACG
EVL-F	CTTCCGTGATGGTCTACGATG
EVL-R	TGCAACTTGACTCCAACGACT

### Statistical Analysis

To investigate the relationship between *ESR2* expression and survival rate in SRCGC, Kaplan–Meier plots were generated online^1^. Accordingly, we drew the survival curve using GraphPad Prism 7.0. Other clinicopathological data were analyzed using SPSS version 18.0 (SPSS Inc., United States). The chi-square test, chi-square test with continuity correction, Fisher’s exact test, and Student’s *t*-test were used to analyze the association between *ESR2* expression and the clinicopathological characteristics in the 42 patients with SRCGC. The differences were statistically significant when *P* < 0.05.

### Cell Proliferation

The cell proliferation assays were performed using a BeyoClick^TM^ EdU Cell Proliferation Kit with Alexa Fluor 488 (BeyoTime). The pre-treated cells were seeded in 24-well plates at 5 × 10^4^ cells per well and cultured under treatment for 24 h. The staining was performed after that, following the kit instructions. The nuclei were stained with Hoechst 33342 (1:1000, BeyoTime). After washing with PBS, fluorescence was detected using the Inverted Microscope Solution DMi8 S Platform (Leica, Germany) and obtained via Leica Application Suite X.

### Lentivirus Transfection

NUGC-4 cells overexpressing ERβ were constructed using lentiviral vector (GeneChem, Shanghai, China). The transfection was performed strictly according to the manufacturer’s instructions. After co-culture with lentivirus for 3 days, the cells were treated with puromycin to eliminate the untransfected cells. Transfection was confirmed in the surviving cells by qRT-PCR. The transfected cells were then used in further experiments.

### Small Interfering RNA (siRNA) Transfection

The following siRNAs were synthesized by Guangzhou RiboBio Tech Co., Ltd., (Guangzhou, China): sense, 5′-GAUUAUAUUUGUCCAGCUATT-3′; and antisense, 5′-UAGCUGGACAAAUAUAAUCTT-3′.

After counting, 5 × 10^5^ NUGC-4 cells were seeded in 6-well plates and reached 30–40% density per well when attached to the bottom. Then, the tumor cells were transfected using siRNA pre-treated with INTERFERin (Polyplus-transfection, France). Transfection was confirmed by qRT-PCR and the cells were used in the following assays.

### Pseudopodia Formation Detection

To investigate how ERβ and its downstream signaling pathways could affect cytoskeleton remodeling and pseudopodia formation, NUGC-4 cells were seeded on UV-treated slides for 24 h, and treated in culture. The cells were then fixed with 4% paraformaldehyde and permeated with 0.3% Triton X-100. F-actin, a significant pseudopodia component, was marked using phalloidin labeled with Alexa Fluor 555 (A34055, Invitrogen, United States). Cortactin and DAPI (4′,6−diamidino−2−phenylindole) labeling was then performed following the instructions. The slides were mounted with ProLong^TM^ Gold Antifade Mountant (P36930, Life Technologies, United States). Confocal micrographs were obtained using an FV3000 confocal laser scanning microscope (Olympus, Japan) under control of supporting software.

## Results

### Loss of ERβ Expression Is Correlated With Poor Prognosis of SRCGC Patients

**FIGURE 1:**
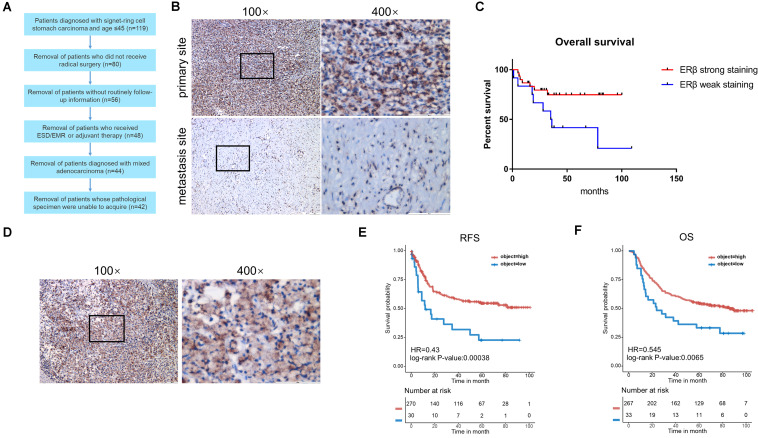
Clinicopathological analysis and ACRG cohort analysis. **(A)** Flow chart of patients screening. **(B)** Immunohistochemical staining with anti-ERβ antibody on primary tumor site and distant metastasis. **(C)** Kaplan-Meier survival curve comparing months of survival in ERβ strong staining and weak staining signet ring cell gastric carcinoma (SRCGC) patients. **(D)** Immunohistochemical staining with anti-ERβ antibody to show the distribution of ERβ. **(E,F)** Kaplan-Meier 5-year relapse-free survival (RFS) and overall survival (OS) curve comparing months of survival in ERβ high-expression and low-expression group was drawn after ACRG data analysis. HR, Hazard Ratio. ACRG, Asian Cancer Research Group.

Figure 1A shows the patients selected into the study. Using IHC, the 42 patients with SRCGC were divided into ERβ^high^ and ERβ^low^ groups. The difference in clinicopathological characteristics between the two groups was explored using statistical analysis, and the results are listed in Table 2. ERβ^high^ patients tended to have lower AJCC (American Joint Committee on Cancer) stage and T stage, indicating that ERβ may play a role in preventing tumor metastasis. Meanwhile, patients with distant metastasis had many more ERβ^low^ signet ring cells in the metastasis sites than in the primary tumor (Figure 1B). At the same time, a survival curve was drawn according to the statistics (Figure 1C). Interestingly, Matsuyama et al. found that ERβ, as a nuclear receptor, can be expressed not only in the nucleus, but also in the cytoplasm (Wang et al., 2007), and our IHC results showed that ERβ could be completely ectopically expressed in the cytoplasm instead of in the nucleus (Figure 1D). Patients with this pathological characteristic were also believed to have cancer metastasis risk. To avoid stochastic error caused by the limited sample capacity, we analyzed the ACRG cohort, which consisted of 300 primary gastric cancer tissues from patients had undergone surgical treatment at Samsung Medical Center (Seoul, South Korea) in 2004–2007. The results showed that high ERβ expression could be linked to higher 5-year overall survival and 5-year relapse-free survival rates (Figures 1E,F).

**TABLE 2:** Clinicopathological information of patients with SRCGC.

Variable	ERβ expression		*P*-value
		
	Strong (*n* = 30)	Weak (*n* = 12)	
Age at diagnosis (years)	35.33 ± 6.697	38.50 ± 6.053	
Gender			0.470
Male	13	6	
Female	17	6	
Tumor size (cm)			0.149
<5	24	7	
≥5	6	5	
AJCC stage			0.045*
I	8	0	
II	9	3	
III	10	7	
IV	3	2	
T stage			0.043*
T1	10	0	
T2	3	0	
T3	7	6	
T4	10	6	
N stage			0.716
N0	11	1	
N1	2	5	
N2	5	2	
N3	12	4	
M stage			0.552
M0	27	10	
M1	3	2	
Nervous invasion			0.280
Positive	20	10	
Negative	10	2	
Venous invasion			0.379
Positive	13	7	
Negative	17	5	
Ki67 expression (%+)			0.688
<50%	12	4	
≥50%	18	8	
Overall survival	74.8%	20.8%	0.028*

*AJCC, American Joint Committee on Cancer, 8th edition, ^∗^*p* < 0.05.*

### ERβ Knockdown Enhances Malignancy of NUGC-4 Cells

**FIGURE 2:**
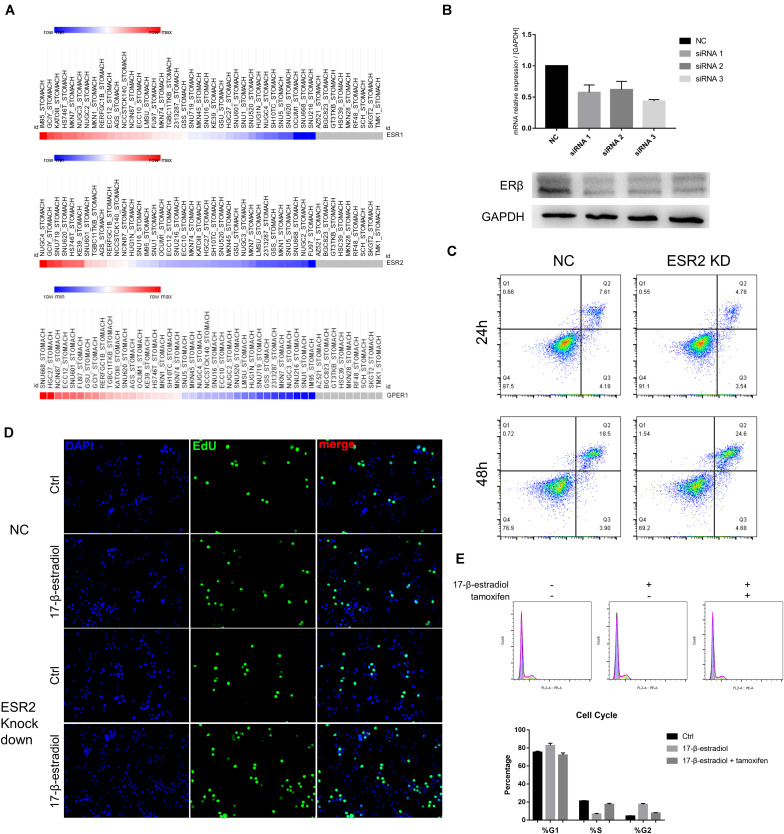
ERβ knockdown was associated with tumor progression. **(A)** Data of mRNA expression was downloaded from CCLE and analyzed on Morpheus. **(B)** Effectiveness of siRNA knockdown was examined by qRT-PCR and Western Blot assays. The ERβ showed double stripe. **(C)** Cell viability of NC and ERβ knockdown NUGC-4 cells was detected after being treated with 1 nM 17-β-estradiol in ultra-low detachment plate for 24 and 48 h. **(D)** Edu assays was performed to detect NC and ERβ knockdown NUGC-4 cell proliferation under different treatment for 24 h. **(E)** Cell cycle analysis of NC and ERβ knockdown NUGC-4 cells under different treatment in ultra-low detachment plate for 24 h. All the *in vitro* experiments were carried out at least 3 times.

Next, to explore whether ERβ expression could play a suppressive role independent of ERα in SRCGC tumor progression, we decided to select ERβ^high^ and ERα^low^ cell lines to avoid the influence of other ERs under estrogen stimulation as much as possible. *ESR1*, *ESR2*, and *GPER1* mRNA expression data of all gastric carcinoma cell lines were downloaded from the Broad Institute Cancer Cell Line Encyclopedia (CCLE^2^) and analyzed on Morpheus^3^. Among these cell lines, we selected as our research tools NUGC-4 cells, instead of KATO III cells, which are more frequently used in research but which express high ERα and low ERβ (Figure 2A). We then used siRNA to knock down *ESR2* expression (ERβ^KD^) and performed qRT-PCR to verify the effect and to choose the most effective knockdown tool (Figure 2B). ERβ^KD^ cells showed higher ability to resist anoikis in an ultra-low attachment environment with the treatment of 17-β-estradiol, and these results could be reversed by tamoxifen, a selected estrogen receptor modulator (Figure 2C). The ERβ^KD^ cells were more sensitive to estrogen and could be stimulated to proliferate (Figure 2D). Cell cycle assays confirmed that estrogen could lead to G2/M arrest (Figure 2E).

### ERβ Over-Expression Inhibits NUGC-4 Cells Malignant Biological Manner

**FIGURE 3:**
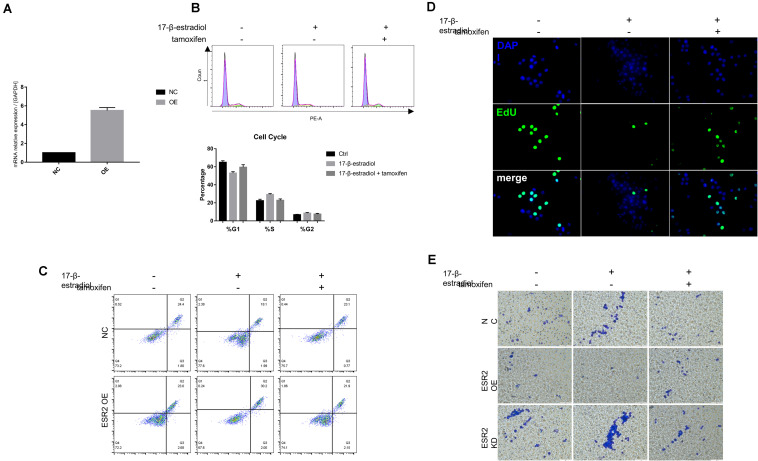
Over-expression of ERβ prevent tumor progression. **(A)** Overexpression of ERβ was examined by qRT-PCR assays. **(B)** Cell cycle analysis of NC and ERβ^OE^ NUGC-4 cells under different treatment in ultra-low detachment plate for 24 h. **(C)** Cell viability of NC and ERβ^OE^ NUGC-4 cells was detected after being treated with 1 nM 17-β-estradiol in ultra-low detachment plate for 48 h. **(D)** Edu assays was performed to detect NC and ERβ^OE^ NUGC-4 cell proliferation under different treatment for 24 h. **(E)** Invasion of NC, ERβ^OE^, and ERβ^KD^ cells was assessed by a Matrigel invasion assay system (magnification x200). All the *in vitro* experiments were carried out at least 3 times.

To explore whether estrogen and tamoxifen treatment could lead to ERβ activation and its function when being activated, ERβ overexpression cells (ERβ^OE^) were constructed using lentiviral vector. The overexpression was also confirmed by qRT-PCR (Figure 3A). We conducted the same assays to confirm that ERβ activation could lead to tumor cell cycle arrest in the S phase (Figure 3B) and tumor sensitivity to anoikis (Figure 3C). Cell proliferation assays proved that estrogen stimulation could suppress ERβ^OE^ cell proliferation (Figure 3D). *In vitro* cell invasion assays showed that ERβ^OE^ tumor cells were difficult to cross the Matrigel with the stimulation of 17-β-estradiol, while tamoxifen-treated ERβ^OE^ cells accelerate tumor invasion. On the contrary, 17-β-estradiol-treated ERβ^KD^ cells gained more ability to cross the Matrigel, and tamoxifen could reverse the phenomenon, indicating that ERβ expression could lead to disabled invasiveness in NUGC-4 tumor cells (Figure 3E).

### ERβ May Inhibit Pseudopodia Formation via the mTOR–Arpc1b/EVL Signaling Pathway

**FIGURE 4:**
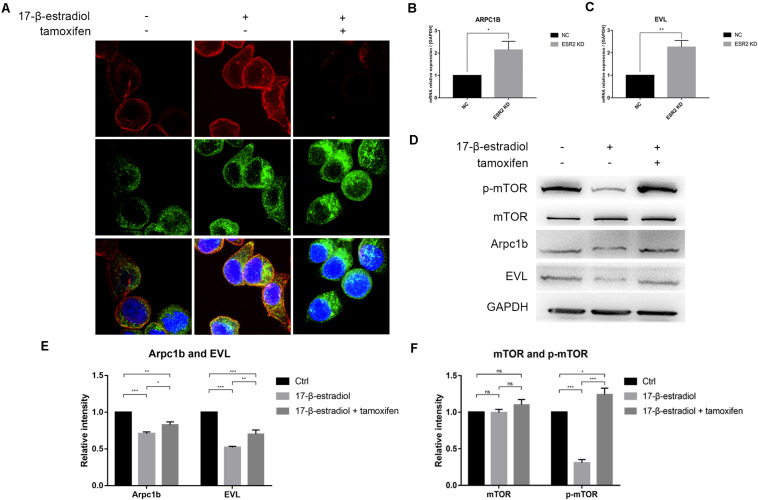
ERβ negatively regulates NUGC-4 invasion by inhibiting pseudopodia formation via mTOR-Arpc1b/EVL pathway. **(A)** Actin and cortactin were stained to reflect ERβ^KD^ NUGC-4 cells pseudopodia formation (Red arrows) and visualized by confocal fluorescence microscopy. Nuclei were stained with DAPI. **(B,C)** ARPC1B **(B)** and EVL **(C)** expression in NC and ERβ^KD^ cells were detected by qRT-PCR assays. **(D)** Western blot analysis of p-mTOR, mTOR, and downstream protein Arpc1b and EVL expression in ERβ^OE^ tumor cells. **(E,F)** Quantification for Arpc1b and EVL **(E)** and mTOR and p-mTOR **(F)** of Western blot assays. *p < 0.05, **p < 0.01, ***p < 0.001.

As ERβ^low^ patients were more likely to have higher T stage in tumor progression, we thought that ERβ could actually play a tumor-suppressive role in SRCGC by inhibiting its invasiveness. Therefore, we performed pseudopodia formation assays, and the results showed, remarkably, that ERβ^KD^ cells could form pseudopodia; tamoxifen treatment significantly suppressed cytoskeleton remodeling (Figure 4A). Previous studies have found that estrogen can inhibit actin-related protein complex subunit 1b (Arpc1b) and Enah/Vasp-like (EVL) expression via ERβ (Kumar et al., 2015; Padilla-Rodriguez et al., 2018). Due to their importance in actin regulation, we conducted qRT-PCR and confirmed that both Arpc1b and EVL were downregulated when ERβ^KD^ cells were stimulated by estrogen (Figures 4B,C). Immunoblotting showed that mTOR was inactivated when ERβ^OE^ cells were stimulated with estrogen (Figure 4D) and quantifications were performed correspondingly (Figures 4E,F). Therefore, we believe that loss of ERβ in SRCGC cells might lead to them becoming more malignant and invading into deep tissue more easily via the mTOR signaling pathway, and their anoikis-resistant characteristic finally aids their metastasis from the primary lesion to distant areas.

## Discussion

Gastric carcinoma, one of the most malignant tumors accounting for nearly one-fifth of cancer incidence and mortality in China, was thought to have no difference in terms of morbidity and mortality between male and female patients (Chen et al., 2016). However, its strong tendency for heterogeneity has led to the possibility that common adenocarcinoma may conceal the sex difference in some unique tumors. SRCGC is one such unique tumor, and has a typical histology characteristic of mucin-filled large vacuoles that occupy most of the cell area, while the nucleus is displaced to the margin, leading to the signet ring resemblance. Due to its poor differentiation, it has worse prognosis than non-SRCGC in the progressive stage (Kao et al., 2019).

Hence, our research team attempted to determine the difference in the clinicopathological characteristics of patients with SRCGC. Experienced doctors at our institution found that, among the younger patients, female patients were more likely to have SRCGC compared to non-SRCGC, and previous studies have also shown an increased proportion of female patients with SRCGC compared with patients with non-SRCGC (Taghavi et al., 2012; Kao et al., 2019). It has been widely acknowledged that estrogen and its receptors are crucial for tumorigenesis and tumor development (Liang and Shang, 2013). Therefore, it has been speculated that the physiological periodic estrogen endocrine or ER can be stimulatory or inhibitory factors in SRCGC tumorigenesis and development. However, although research has indicated that older men are more likely to have intestinal-type gastric adenocarcinoma (Tanaka et al., 1987; Taghavi et al., 2012), male patients with SRCGC with estrogen levels that are relatively and constantly low compared to female patients also accounted for half of the patients (Taghavi et al., 2012; Kao et al., 2019). Therefore, we then focused on whether changes in the expression level or ectopic expression of its receptor, or even changes in the downstream signaling pathway, play a role instead of estrogen itself.

Among the common ERs, ERβ protein is expressed in gastric cancer cells as described above. Therefore, we choose ERβ as our research object. Although previous research has found that ERβ may show sex-associated differences in MNNG (N-methyl-N′-nitro-N′-nitrosoguanidine)-induced gastric cancer in rats (Wakui et al., 2011), our clinicopathological statistics are not consistent with this finding. This may be attributed to our sample size, as well as the differences between humans and rats. ERβ is expressed in gastric cancer cells at both mRNA and protein level, and loss of ERβ was correlated to decreased differentiation (Takano et al., 2002; Xu et al., 2010). In SRCGC, we found that ERβ was expressed in these low-differentiation cells, but the expression level was negatively related to the depth of tumor invasion. For some samples, the receptor was not detected in the nucleus, but in the ectopic area. It has been reported that ectopic expression of ERβ in the cytoplasm is that of its isoform (Chakravarty et al., 2014; Fu et al., 2018). We did not analyze whether ectopic expression can influence tumor progression, and previous studies have shown that ectopic ERβ may be linked to tumor development (Schuler-Toprak et al., 2018). As one of the most important functions of ERβ is to regulate transcription, its ectopic expression is not believed to be correctly regulatory and cannot correctly perform its anti-tumor function.

The main ERs are ERα, ERβ, and GPER1. ERα promotes tumor progression (Shaaban et al., 2008; Gao et al., 2018), while the role of GPER1 remains controversial (Zhou et al., 2015; Liang et al., 2017; Chaturantabut et al., 2019). So we decided to use an ERβ^high^, ERα^low^, and GPER1^low^ cell line to avoid interference from the latter two receptors under the limited conditions (Lee et al., 2019). Despite the low expression of ERα and GPER1 in the NUGC-4 cells, it seemed that estrogen benefited tumor cell survival in normal tumor cells. One possible reason for this phenomenon is that estrogen might predominantly bind to ERα to play a pro-tumor role, and we demonstrated that estrogen had a greater tendency to bind to ERβ and play an anti-tumor role with the overexpression of ERβ. Our findings show that ERβ can negatively affect tumor progression by inhibiting tumor proliferation and invasiveness.

Tamoxifen, as a selective estrogen receptor modulator, has been used in our study since it has been proved to inhibit ER-negative SRCGC cell line proliferation (Hosoya et al., 1999). Another reason why we choose tamoxifen to evaluate its therapeutic effect is that this drug has come into public and is relatively cheap for the patients. Despite this, clinical doctors keep cautious attitude toward its usage because the therapeutic effect of this drug is not clearly verified, and the drug itself is thought to induce acute spasmolytic polypeptide-expressing metaplasia (SPEM) in normal stomach (Huh et al., 2012; Radyk et al., 2018). As the result, we have to exactly prove the therapeutic effect of it to the ERβ-negative tumor cells. We applied 1 μM 17-β-estradiol in this study because gastric cancer cells are sensitive to this hormone at this concentration (Kim et al., 2013). Similarly, we used 1 μM tamoxifen following the previous study (Liang et al., 2017). Our study confirms the conclusion as proliferation and invasion of ERβ^KD^ NUGC-4 cells are inhibited, but the reverse takes place in ERβ^OE^ NUGC-4 cells as tamoxifen application is able to promote their proliferation and invasion. Therefore, we think the results may support us to use tamoxifen in clinical treatment for the ERβ-negative SRCGC.

Other studies have also shown that the absence of intestinal ERβ could lead to enhanced tumorigenesis, as it could not perform its suppressive function for colon inflammation (Hases et al., 2020). In the present study, we focused on the mechanism of the regulatory function of ERβ in invasiveness, as the *in vitro* results corresponded with the clinicopathological findings. Estrogen can downregulate the expression of Arpc1b and EVL, which are important cytoskeleton remodeling components, by activating ERβ(Kumar et al., 2015). We performed pseudopodia formation staining, and showed that ERβ^KD^ cells were stimulated to form pseudopodia, and that tamoxifen could reverse this, which might further show that tamoxifen could be used in treatment to ERβ-negative SRCGC. Western blotting and qRT-PCR further showed that mTOR–Arpc1b/EVL could be involved in pseudopodia formation.

**FIGURE 5:**
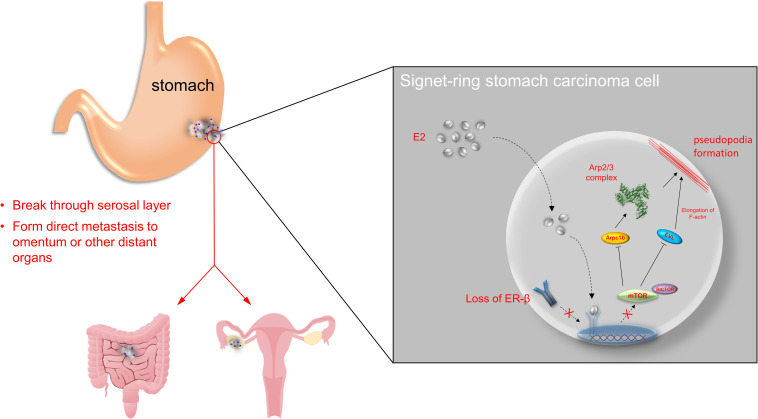
Overview of the study. ERβ is found to be expressed in stomach adenocarcinoma in both mRNA and protein levels. Normally, estrogen binds to ERβ in nuclear and suppress mTOR-Arpc1b/EVL signaling pathway. However, tumor progression may be along with loss of ERβ, which accelerates SRCGC invasion and distant metastasis.

In conclusion, our findings reveal that ERβ may participate in SRCGC progression by inhibiting cell proliferation and invasiveness via the mTOR–Arpc1b/EVL signaling pathway (Figure 5). However, as the 17-β-estradiol used in this study is not selective and can activate all ERs, future studies should determine its clinical significance by using an ERβ-specific agonist to prove the anti-tumor function of ERβ. Besides, the loss of ERβ can disable the protective downstream signaling pathway, so further exploration to identify a specific target in the pathway may help induce the anti-tumor effect without the presence of ERβ.

## Data Availability Statement

Publicly available datasets were analyzed in this study. This data can be found here: https://www.ncbi.nlm.nih.gov/geo/query/acc.cgi?acc=GSE62254.

## Ethics Statement

All materials and their usage in this project were reviewed and approved by the Nanjing Drum Tower Hospital Review Board.

## Author Contributions

WJ, WG, and XX formulated the hypothesis and help design the study. XW, XX, and EX took part in article writing and *in vitro* assays. ZY helped performs ACRG cohort analysis. XL, XS, and SD helped collected the samples. XC helped analyze the clinicopathological statistics. All authors contributed to the article and approved the submitted version.

## Conflict of Interest

The authors declare that the research was conducted in the absence of any commercial or financial relationships that could be construed as a potential conflict of interest.

## Footnotes

1 http://kmplot.com/analysis

2 https://portals.broadinstitute.org/ccle

3 https://software.broadinstitute.org/morpheus/
